# Neuronal Activity Changes the Number of Neurons That Are Synaptically Connected to OPCs

**DOI:** 10.1523/ENEURO.0126-23.2023

**Published:** 2023-10-23

**Authors:** Daniela Moura, Alekhya Parvathaneni, Atehsa Sahagun, Hirofumi Noguchi, Jesse Garcia, Emma Brennan, Robert Brock, Iris Tilton, Lindsay Halladay, Samuel Pleasure, Laura Cocas

**Affiliations:** 1 Biology Department, Neuroscience Program, Santa Clara University, Santa Clara, CA 95053; 2 Neurology Department, University of California, San Francisco, San Francisco, CA 94110

**Keywords:** activity-dependent development, connectivity, gliogenesis, neural glial interactions, synapse formation

## Abstract

The timing and specificity of oligodendrocyte myelination during development, as well as remyelination after injury or immune attack, remain poorly understood. Recent work has shown that oligodendrocyte progenitors receive synapses from neurons, providing a potential mechanism for neuronal-glial communication. In this study, we investigated the importance of these neuroglial connections in myelination during development and during neuronal plasticity in the mouse hippocampus. We used chemogenetic tools and viral monosynaptic circuit tracing to analyze these connections and to examine oligodendrocyte progenitor cells (OPCs) proliferation, myelination, synapse formation, and neuronal-glial connectivity *in vivo* after increasing or decreasing neuronal activity levels**.** We found that increasing neuronal activity led to greater OPC activation and proliferation. Modulation of neuronal activity also altered the organization of neuronal-glial connections: while it did not impact the total number of RabV-labeled neuronal inputs, or the number of RabV-labeled inhibitory neuronal (IN) inputs, it did alter the number of RabV-labeled excitatory neuron to OPC connections. Overall, our findings support the idea that neuronal activity plays a crucial role in regulating OPC proliferation and activation as well as the types of neuronal inputs to OPCs, indicating that neuronal activity is important for OPC circuit composition and function.

## Significance Statement

Neuronal degeneration, traumatic brain injury, and multiple sclerosis all share a common clinical feature: loss of myelin on the axons of neurons, which is critical for accurate and rapid conduction of information throughout the CNS. Understanding the maturation of the glial cells that make myelin is critical to develop therapeutics to restore myelin and treat demyelinating diseases and brain injury. Our work provides insight on the role of neuronal activity in cuing oligodendrocyte progenitor cell (OPC) proliferation, activation, and neuron to glia synapse formation, expanding our growing knowledge about the importance of synaptic interactions between neurons and glia in the brain.

## Introduction

Fast and efficient conduction of electrical nerve impulses happens via saltatory conduction thanks to the insulation of the axons through the myelin sheath (Stämpfli, 1954). Myelination is necessary for the maturation of the neural circuitry required for cognition, complex motor behaviors, and sensory integration. It also regulates the timing of activity in neural circuits and is important for maintaining the health of axons and providing nutritional support (Saab and Nave, 2017). Oligodendrocyte lineage cells are responsible for producing myelin and continue to proliferate and add new myelin throughout the lifespan. In humans, developmental myelination concludes by age 40, after which a secondary phase of myelination, known as adaptive myelination, continues. Changes in myelination occur after motor learning and recovery from injury; reductions in myelination occur after sensory or social deprivation (McKenzie et al., 2014; Liu et al., 2016; Etxeberria et al., 2016; Bechler et al., 2018; Duncan et al., 2018).

Oligodendrocyte progenitor cells (OPCs) are glial progenitor cells that can become myelinating oligodendrocytes; they are one of the largest pools of dividing cells in the postnatal brain. OPCs can produce new oligodendrocytes that remyelinate axons after injury and in demyelinating diseases or injuries such as MS and hypoxia (Wang et al., 2018). OPCs receive synapses from neurons in both white and gray matter and are activated by the neurotransmitters glutamate and GABA from neuronal presynaptic vesicles (Bergles et al., 2000; Lin and Bergles, 2004). They express various ion channels and receptors, including Na^+^, K^+^, and Ca^2+^ voltage-gated channels, as well as AMPA, NMDA, GABAA, and GABAB receptors (Bergles et al., 2000; Kukley et al., 2010; Paoletti et al., 2013; Serrano-Regal et al., 2020; Moura et al., 2022). The expression of these channels and receptors is developmentally regulated, peaking early in postnatal development at a time window consistent with developmental myelination (Spitzer et al., 2019). These data suggest that neuronal-glial synapse formation is important for myelination. Electrophysiological recordings in hippocampal slices determined the functional nature of these synapses showing that OPCs receive both excitatory and inhibitory inputs, and that the neuronal inputs evoke small postsynaptic currents (EPSCs or IPSCs, respectively) in OPCs in the hippocampus (Bergles et al., 2000; Lin and Bergles, 2004). Electron microscopy confirmed at the level of individual synapses the presence of presynaptic densities adjacent to OPC structures that exhibit many of the features of a postsynaptic structure (Bergles et al., 2000). Others have found neuron to OPC connections in multiple regions of the cortex, but no changes in connectivity after alterations of sensory inputs (Mount et al., 2019). Our paper builds on these findings by investigating changes in neuron to OPC connectivity after altering neuronal activity, with the aim of gaining a better understanding of the mechanisms that may initiate myelination.

Several lines of evidence support the idea that neuronal activity influences OPC development and myelination (for review, see Gallo et al., 1996; Yuan et al., 1998; Gudz et al., 2006; Bergles et al., 2010; also see Hines et al., 2015). Chemogenetic manipulation to increase neuronal activity leads to an increase in OPC proliferation and differentiation, as well as increasing the probability of an axon being myelinated, and results in thicker myelin on stimulated axons (Mitew et al., 2018). Decreasing neuronal activity can decrease the probability of myelination, indicating that myelination is activity-dependent and that neuronal activity is instructive for myelination. This process is regulated during development, as juvenile mice exhibit a greater increase in OPC proliferation in response to increased neuronal activity compared with adult mice (Gibson et al., 2014; McKenzie et al., 2014; Mitew et al., 2018). Blocking sensory inputs from neurons in the retina during the critical period of visual sensory input results in a decrease in myelination of the optic tract (Etxeberria et al., 2016). Because of these earlier findings, we chose to focus on juvenile mice, when the initial period of myelination is still occurring. We sought to determine whether neuronal activity directly changes OPC proliferation or activity, whether neuronal activity modulation altered the total neuron to OPC connectivity, and finally, the role of neuronal activity on the types of neuronal inputs to OPCs. Previous research found varying results regarding changes to OPC proliferation and myelination when increasing or decreasing neuronal activity (Mitew et al., 2018). We aimed to expand this research direction, using chemogenetics to investigate whether increasing or decreasing neuronal activity during the period of enhanced developmental synapse formation and myelination would impact the connectivity between neurons and OPCs. Our findings demonstrate that altering neuronal activity increases OPC proliferation, and that increasing (but not decreasing) neuronal activity increases OPC activation and the number of excitatory neuron to OPC connections, discussed further below.

## Materials and Methods

### Animals

All animal procedures were approved by the Institutional Animal Care and Use Committees at Santa Clara University and the University of California, San Francisco. Pdgfra-CreERT2 driver mice were crossed to ROSA-tTA and pTRE-Bi-G-TVA mice and the resulting litters were genotyped to generate Pdgfra-CreERT2; ROSA-tTA; pTRE-Bi-G-TVA mice (Yasuda and Mayford, 2006; Kang et al., 2010; Han et al., 2013). Following tamoxifen-induced gene recombination, Pdgfra-CreERT2; ROSA-tTA; pTREBi-G-TVA mice express the avian envelope protein TVA and the rabies glycoprotein GRab under tetracycline control, only in OPC cells expressing Pdgfra. This allows for OPC-specific targeting of the viral proteins necessary for infection (TVA) and spread (GRab). Saline or clozapine was delivered intraperitoneally every day for 7 d; clozapine was delivered at a dose of 0.1 mg/kg. Tamoxifen was delivered at a dose of 0.5 mg/40 g via intraperitoneal injections, spaced over 7 d with 24 h between the last dose and either viral injection or euthanasia. EdU was delivered intraperitoneally every day for 7 d at a dose of 10 mg/kg.

### Viruses

Two avian envelope protein pseudotyped (EnvA) G-deleted mutant rabies viruses that expressed either a red fluorescent reporter [(EnvA)SADΔmCherry] or a green fluorescent reporter [(EnvA)SADΔGFP] were amplified and pseudotyped from stock viruses (kind gift of the Callaway lab; Salk Institute) following their established protocol (Wickersham et al., 2010). Each virus was titered on mammalian 3T3 cells to confirm that the virus expressed the EnvA protein, and to determine the viral titer: 1 × 10^9^ IU ml^−1^ was reached for each round of production.

An AAV virus expressing pAAV-hSyn-hM4D(Gi)-mCherry (AAV8), which would lead to decreased activity in projection neurons of the cortex, was sourced from Addgene (50475), as was an AAV virus expressing pAAV-hSyn-hM3D(Gq)-mCherry (AAV8), which would lead to increased activity in projection neurons of the cortex (Addgene, 50474).

### Viral circuit tracing by brain region

**Figure 1.**
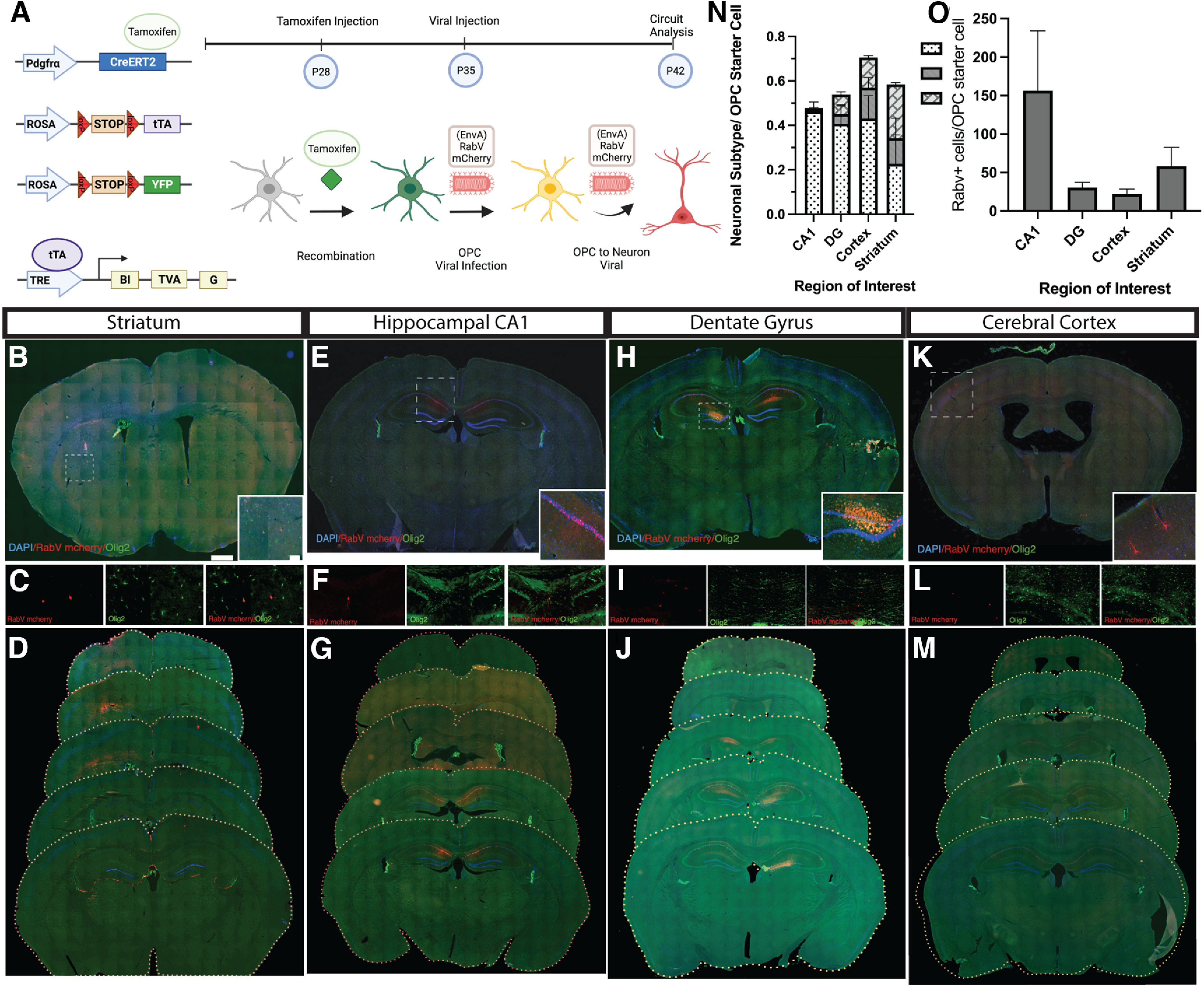
Neuron to OPC circuits have diverse patterns of connectivity in the forebrain. ***A***, We combined attenuated rabies virus with a Cre-loxP based system using the Pdgfra-^CreERT2^ driver mouse crossed to ROSA^tTA^; pTRE-Bi-G-TVA mice, inducing recombination in OPCs using tamoxifen. These mice now express the avian envelope protein TVA and the rabies glycoprotein GRab. This allows us to target only OPCs with the viral proteins necessary for infection (TVA) and spread (GRab) along with a label of recombined OPCs. We target OPCs with a viral injection of avian envelope protein pseudotyped deletion mutant rabies virus (EnvA)RabV that expresses a red fluorescent reporter (mCherry), prepared as previously shown (Wickersham et al., 2010), and counterstain all sections with Olig2. Striatum (***B–D***), CA1 (***E–G***), dentate gyrus (***H–J***), and somatosensory cortex (***K–M***) targeting of neuron to OPC circuits using monosynaptic viral circuit tracing. Pdgfra-CreERT2; YFP; tTA; G-TVA mice injected at P60 with (EnvA)RabV mcherry after 5 d of tamoxifen treatment. ***C***, ***F***, ***I***, ***L***, Neurons (labeled in red) are presynaptically connected to each OPC population (labeled with both red and green). ***N***, Quantification of: total neuronal (Neun+) inputs, PV+ (parvalbumin+) interneuron inputs, and SST+ (somatostatin+) interneuron inputs connected to OPCs in CA1, dentate, striatum, and somatosensory cortex. For Neun: *N* = 4 (CA1, mean = 0.4632, SEM = 0.4214; DG, mean = 0.4074, SEM = 0.08; Cortex, mean = 0.4300, SEM = 0.1039; STR, mean = 0.2270, SEM = 0.1302). For PV: *N* = 4 (CA1, mean = 0.1143, SEM = 0.0074; DG, mean = 0.0441, SEM = 0.0409; Cortex, mean = 0.1394, SEM = 0.0492; STR, mean = 0.1156, SEM = 0.0900). For SST: *N* = 4 (CA1, mean = 0.0038, SEM = 0.0000; DG, mean = 0.0865, SEM = 0.0127; Cortex, mean = 0.13 642, SEM = 0.0088; STR, mean = 0.2419; SEM = 0.0816). ***O***, Quantification of number of RabV+ inputs per OPC starter cell in each of the brain regions in ***B–M***. *N* = 4 (CA1, mean = 156.3182, SEM = 77.68; DG, mean = 30.6061, SEM = 6.63; Cortex, mean = 22.0000, SEM = 6.5320; STR, mean = 58.33, SEM = 24.333). Scale bar: 50 μm, 500 μm. Error bars are standard error of the mean. *Figure Contributions*: Alekhya Parvathaneni and Atehsa Sahagun collected data, Iris Tilton, Alekhya Parvathaneni, and Laura Cocas analyzed data, Laura Cocas and Robert Brock made the figure.

Pdgfra-CreERT2; ROSA-tTA; pTREBi-G-TVA; ROSA-YFP (*N* = 4–6/group, counterbalanced for sex) were dosed with tamoxifen (dose: 2 mg/25 g body weight) via intraperitoneal injection for 5 d starting from postnatal day (P)25. Twenty-four hours after the last tamoxifen injection, mice were stereotaxically injected with 300 nl of EnvA pseudotyped G-deleted rabies virus expressing mCherry (EnvA ΔGRabV-mCherry, 300 nl). Briefly, we used an attenuated rabies virus that expresses red fluorescent protein, making it possible to analyze retrograde (presynaptic) monosynaptic connections *in vivo*. By combining attenuated rabies virus with a Cre-loxP based system, we examined neuronal input onto OPCs using the Pdgfra-CreERT2 driver mouse crossed to ROSA-G-TVA mice and we induced recombination in OPCs using tamoxifen. These mice now expressed the avian envelope protein TVA and the rabies glycoprotein GRab. This allows us to target only OPCs with the viral proteins necessary for infection (TVA) and spread (GRab). We targeted OPCs with a viral injection of avian envelope protein pseudotyped deletion mutant rabies virus (EnvA)RabV that expresses a red fluorescent reporter (mCherry), prepared in our lab using standard protocols and as we have previously published (Wickersham et al., 2010; Cocas et al., 2016). We then examined the neurons (labeled in red) that are presynaptically connected to each OPC population (labeled with both red and green; Fig. 1*A*). We stained for immunomarkers of neuronal subtypes and scanned whole brains for imaging. We then quantified the numbers, types, and locations of cells based on this approach, which allowed us to analyze regional differences in neuron to OPC circuits.

Stereotaxic injections occurred in CA1 of the hippocampus (*X* 1.1, *Y* −1.7, *Z* 1.4), the dentate gyrus (*X* 2.1, *Y* −2.7, *Z* 2.9), striatum (*X* 2.5, *Y* 0, *Z* 2.6), or cortex (*X* 0.6, *Y* −1.5, *Z* 1.0) using a Kopf sterotaxic rig. Following injection, mice recovered and were placed in group housing for a period of 7 d to allow for viral expression, after which they were killed and the brains were collected and processed for histology. Sections were stained with Olig2, NeuN, parvalbumin (PV), and somatostatin (SST), and the number of RabV+ cells co-labeled with these stains was quantified as was the total number of RabV+ cells.

### Neuronal activity modulation

ROSA-tTA; pTREBi-G-TVA; ROSA-YFP mice (*N* = 4/group, counterbalanced for sex) were stereotaxically injected in the hippocampus with an AAV virus expressing hSyn-hM4D(Gi)-mCherry (400 nl, *X* 1.1, *Y* −1.7, *Z* 1.4), or one expressing hSyn-hM3D(Gq)-mCherry (400 nl, *X* 1.1, *Y* −1.7, *Z* 1.4) between P8 and P12, and returned to group housing for two weeks to allow for viral expression. Mice were injected with intraperitoneal injections of EdU (0.1 mg/25 g) and intraperitoneal injections of either saline or clozapine (0.1 mg/kg) for 7 d starting from P25. Twenty-four hours after injection, mice were killed, and the brain was collected and processed for histology. Sections were stained with Ctip2 and cfos to confirm viral cell infection specificity in neurons. Additional sections were stained with Olig2 and Cfos, and the number of CFos+/Olig2+ cells were quantified for each condition.

### Viral circuit tracing following neuronal activity modulation

Pdgfra-CreERT2; ROSA-tTA; pTREBi-G-TVA; ROSA-YFP (*N* = 4–6/group, counterbalanced for sex, were stereotaxically injected in the hippocampus with an AAV virus expressing hSyn-hM4D(Gi)-mCherry (Adgene; 400 nl, *X* 1.1, *Y* −1.7, *Z* 1.4), one expressing hSyn-hM3D(Gq)-mCherry (Adgene; 400 nl, *X* 1.1, *Y* −1.7, *Z* 1.4), or one expressing hSyn-mCherry (Adgene) between P8 and P12, and returned to group-housing for two weeks to allow for viral expression. After two weeks, mice were administered tamoxifen (dose: 1 mg/25 g body weight) via intraperitoneal injection for 5 d as well as an intraperitoneal injection of clozapine (0.1 mg/kg) or saline for 7 d. Twenty-four hours after the last clozapine or saline injection, mice were injected with EnvA ΔGRabV-GFP (300 nl, *X* 1.1, *Y* −1.7, *Z* 1.4), allowed to recover, and returned to housing. 7 d after rabies injection, mice were killed and the brains were collected and processed for histology. Sections were stained with Ctip2, Olig2, or parvalbumin/somatostatin, and the number of RabV+ cells co-labeled with these stains was quantified as was the total number of RabV+ cells.

### *In vivo* electrophysiology

A microelectrode array (4 × 4 35-μm electrodes) with 150-μm row and electrode spacing (Innovative Neurophysiology) was unilaterally (right hemisphere) targeted to the dHPC (array center: *X* 1.1, *Y* −1.7, *Z* 1.4) and affixed to the skull with dental cement. One week after surgery, mice were habituated to the recording tether for 1 h/d for 3 d in their home cage before recordings.

*In vivo* electrophysiological recordings began two weeks after stereotaxic injection with either hSyn-hM4D(Gi)-mCherry (400 nl, *X* 1.1, *Y* −1.7, *Z* 1.4) hSyn-hM3D(Gq)-mCherry (400 nl, *X* 1.1, *Y* −1.7, *Z* 1.4) and consisted of 120-min sessions in the home cage. After 10 min of drug-free baseline recording, mice were injected with saline or clozapine (0.1 mg/kg, i.p.) and returned to the home cage for the remainder of the recording session. Electrophysiological recordings were acquired using SpikeGadgets main control unit and Trodes software (SpikeGadgets), using 16-channel digitizing head-stages sampled at 30 kHz. Spike sorting was conducted manually using Offline Sorter (Plexon Inc.) and analyzed using NeuroExplorer (Nex Technologies; Halladay and Blair, 2015; Glover et al., 2020).

On completion of testing, mice were anesthetized with 2% isoflurane and a current stimulator (S48 Square Pulse Stimulator, Grass Technologies) delivered 2 s of 40-μA DC current through each electrode to make a small marking lesion. Twenty-four hours later, mice were overdosed via 150 mg/kg intraperitoneal Euthasol (Henry Schein Medical) and perfused intracardially with PBS followed by 4% paraformaldehyde (PFA). Brains were left in 4% PFA overnight, then transferred to a 30% sucrose PBS solution for cryoprotection. Coronal sections (50 μm thick) were cut on a cryostat (Leica Biosystems Inc) and mounted onto slides. Tissue was stained with DAPI (Sigma-Aldrich) and imaged using a Keyence BZ-X800 fluorescence microscope (Keyence Corporation of America). Only single units with placements confirmed to be in the dHPC were included in the analysis.

To determine whether units altered their firing rate following clozapine administration, data during 60 s bins following the injection were *z* score normalized to a 10-min baseline period just before injection. For individual units, time bins with a *z* score of >|1.96| were considered significantly different from baseline (*p* < 0.05; Fig. 1*J*, color maps). Results from a one-way ANOVA confirmed that the unit firing rate was significantly dependent on virus type (*F*_(2,357)_ = 330.23, *p* < 0.0001). Planned *post hoc* comparisons of averaged viral group unit responses (independent samples *t* tests) revealed that compared with the control group, units recorded in mice injected with hM3Dq and hM4Di viral constructs displayed significantly greater or lesser, respectively, average normalized firing rates during the recording session, beginning 15 and 9 min after clozapine administration (*p* < 0.01). (Each significant bin is denoted by orange/blue lines at top of right panel graph.)

### Tissue processing and sectioning

Mice were killed via transcardial perfusion under terminal anesthesia with sodium pentobarbital (250 mg/kg). Anesthetized mice were pinned and visualization of the heart was achieved following removal of the rib cage, after which a 21-gauge needle was inserted into the left ventricle, the vena cava snipped, and 10 ml of ice-cold PBS run through the line, followed by 20 ml of 4% paraformaldehyde. After perfusion, the brain was harvested and placed at 4°C in 4% PFA for 2 h, after which it was moved to 15% sucrose overnight, and then cryoprotected in 30% sucrose overnight. The brain was then embedded in O.C.T. compound (Sakura Finetek) on dry ice and stored at −80°C until sectioned into 25-μm slices on a CM1860 Leica cryostat (Leica Microsystems).

### Immunohistochemistry

After sections were washed thrice with PBST for 10 min at a time, they were incubated in blocking buffer (10% lamb serum, 0.33% Triton X-100, 0.005% sodium azide, and 1:50,000 DAPI in PBS) for 1 h, after which they were incubated in primary antibody for 4 h at room temperature. Following three washes with PBST, sections were then incubated in secondary antibody for 1 h, followed by three washes in PBST, and mounted using Fluoromount-G mounting medium (Thermo Fisher Scientific). Stained sections were imaged using a Zeiss Axioscanner, and representative images for each condition were collected using a Zeiss 710 confocal microscope. The following primary antibodies were used: anti-Olig2 Rb (Abcam ab109186); anti-Olig2 Ms (Millipore MABN50), anti-parvalbumin Ms (Millipore MAB1572), anti-somatostatin Rb (Invitrogen PA585759), anti-Ctip2 rat (Abcam AB18465), anti-CFos Rb (Synaptic Systems 226008), anti-gephyrin Ms (Synaptic Systems 147021), anti-SAP97 Rb (Thermofisher PA1-741), and anti-NeuN Rb (Thermofisher PA5-78499). All secondary antibodies were Alexa Fluor secondary antibodies from Jackson ImmunoResearch, raised in goat, and conjugated to 488, 546, or 633 fluorophores as needed.

### EdU staining

After sections were washed thrice with PBST for 10 min at a time, after which they were stained with EdU using the Click-iT EdU Imaging kit with Alexa Fluor 647 Azide (ThermoFisher, C10337) according to manufacturer instructions. Following EdU staining, slides were incubated in blocking buffer (10% lamb serum, 0.33% Triton X-100, 0.005% sodium azide, and 1:50,000 DAPI in PBS) for 1 h, then immunostained overnight with Olig2. Slides were washed in PBST three times and mounted using Fluoromount-G mounting medium. Representative images for each condition were collected using a Zeiss 710 confocal microscope. The number of EdU+/Olig2+ cells in the hippocampus were quantified for each condition.

### Data collection and statistical analyses

Sections were analyzed to manually quantify the number of co-labeled cells by category. Zeiss Zen lite was used to visualize the image files from the Zeiss Axioscanner for quantification, and Fiji was used to visualize and count the cells in the image files from the Zeiss 780 Confocal Microscope. All schematics were created with BioRender.

For every animal in the viral circuit tracing experiments, we sectioned the entire forebrain in 30-μm sections and counted the total number of RabV+ cells without estimation.

Statistical analyses were performed, first testing for normality using the Shapiro–Wilk test. In instances where the distribution of the data was non-Gaussian, nonparametric analyses were completed using the Mann–Whitney test. In instances where the distribution was normal, unpaired *t* tests were completed for the Gq and Gi conditions separately. The exception is the Edu dataset, where the addition of a control group using an hSyn-mCherry AAV and clozapine treatment necessitated a one-way ANOVA and a planned *post hoc* Sidak’s multiple comparisons test to determine the significant pairwise comparisons between control, Gq, and Gi conditions. Descriptive statistics are included in all figure legends. All data were analyzed and plotted using GraphPad Prism version 8.0.0.

## Results

### Differential neuron to OPC connectivity

In light of previous work by Spitzer and colleagues (2019), we hypothesized that the connections between neurons and OPCs would have regional differences, with different brain regions featuring unique patterns of connectivity. Specifically, we anticipated that these differences would manifest in terms of the cytoarchitecture of the connections, the number of connections, and the specific types of presynaptic inputs onto OPCs. Given the neuron to OPC connections are transient and are lost when OPCs differentiate into oligodendrocytes (De Biase et al., 2010), we used an inducible Pdgfra-Cre mouse to label OPCs during postnatal development, at the peak of OPC differentiation and myelination, when there would likely be large numbers of neuron to OPC connections. We aimed to explore the diversity of these connections among different regions of the forebrain with unique cytoarchitecture, circuitry, and myelin patterns. Specifically, we targeted the cerebral cortex, a six layered gray matter dense structure, the CA1 and DG of the hippocampus, a three layered dense structure of gray matter, the striatum, a region that contains a higher ratio of white matter to gray matter than the other two structures, and the corpus callosum, a white matter rich region. Our goal was to determine whether each of these regions had unique neuron to OPC connections, or whether there were common features among the forebrain structures. By using a Cre-ER mediated system combined with tTA-dependent G and TVA, we were able to specifically target OPCs using a deletion mutant rabies virus. This strategy allowed us to have low numbers of starter OPCs, to ensure that all neurons were directly connected and no viral or genetic leakage was present (Fig. 1*A*; data not shown). This sparse labeling was intentional: too many starter cells can make it challenging to determine the number of inputs. We confirmed that in the absence of G and TVA, the (EnvA) pseudotyped rabies virus did not infect any cells, and also crossed the Pdgfra-Cre-ERT2 to a reporter to confirm that all rabies infected starter cells were OPCs (data not shown). Postnatal administration of tamoxifen was employed to further reduce the chance of recombination in any neuronal starter cells.

Representative images of labeled presynaptic neurons from the somatosensory cortex, corpus callosum, CA1 of the hippocampus, and dentate gyrus illustrate the diverse patterns of neuron to OPC connections (Fig. 1*B–M*). Striatal targeting is mainly limited to local striatal projection neuron inputs with rare contralateral connections or inputs outside of the striatum (Fig. 1*B–D*). CA1 OPCs receive many ipsilateral and contralateral inputs from hippocampal pyramidal neurons (Fig. 1*E–G*). DG targeting revealed CA1 and CA3 neuronal inputs (Fig. 1*H–J*). Cortical inputs to OPCs are primarily pyramidal neurons with few contralateral inputs, and often extend across several layers of the cortex (Fig. 1*K–M*). The patterns of these connections were consistent with their local circuitry: cortical connections were often columnar, with inputs stretching across many layers of the cortex; striatal connections were largely nuclear, with scattered inputs within the caudate and putamen; inputs to CA1-localized OPCs were from many neurons throughout the stratum pyramidale of CA1 and the stratum lucidum of CA3; DG inputs to OPCs were made up of both local granule cells and mossy cells as well as occasional neurons in the entorhinal cortex (Fig. 1*B–M*). We also examined whether inhibitory neurons (INs) made inputs onto OPCs in these different regions (Fig. 1*N*). Interestingly, there were regional differences in the number of neuronal inputs onto OPCs, with the OPCs in the hippocampus receiving more neuronal inputs, compared with the striatum and cortex (Fig. 1*N*). We found that the hippocampal CA1 had the fewest PV+ or SST+ inhibitory neuron (IN) synapses onto OPCs, followed by the DG. The cortex had many more INs connected to OPCs than in the hippocampus, and there were more PV+ INs than SST INs in the cortex, whereas in the striatum there were more SST+ INs than PV+ INs. Finally, we found regional differences in the total number of neuron to OPC synapses, which were much higher in the CA1 of the hippocampus compared with the other regions. The diversity of connectivity between neurons and OPCs in the forebrain, as well as the high level of neuronal inputs onto each OPC in the hippocampus, led us to focus our future experiments on the CA1 region of the hippocampus.

### Manipulation of neuronal activity to test changes in OPC development and function

**Figure 2.**
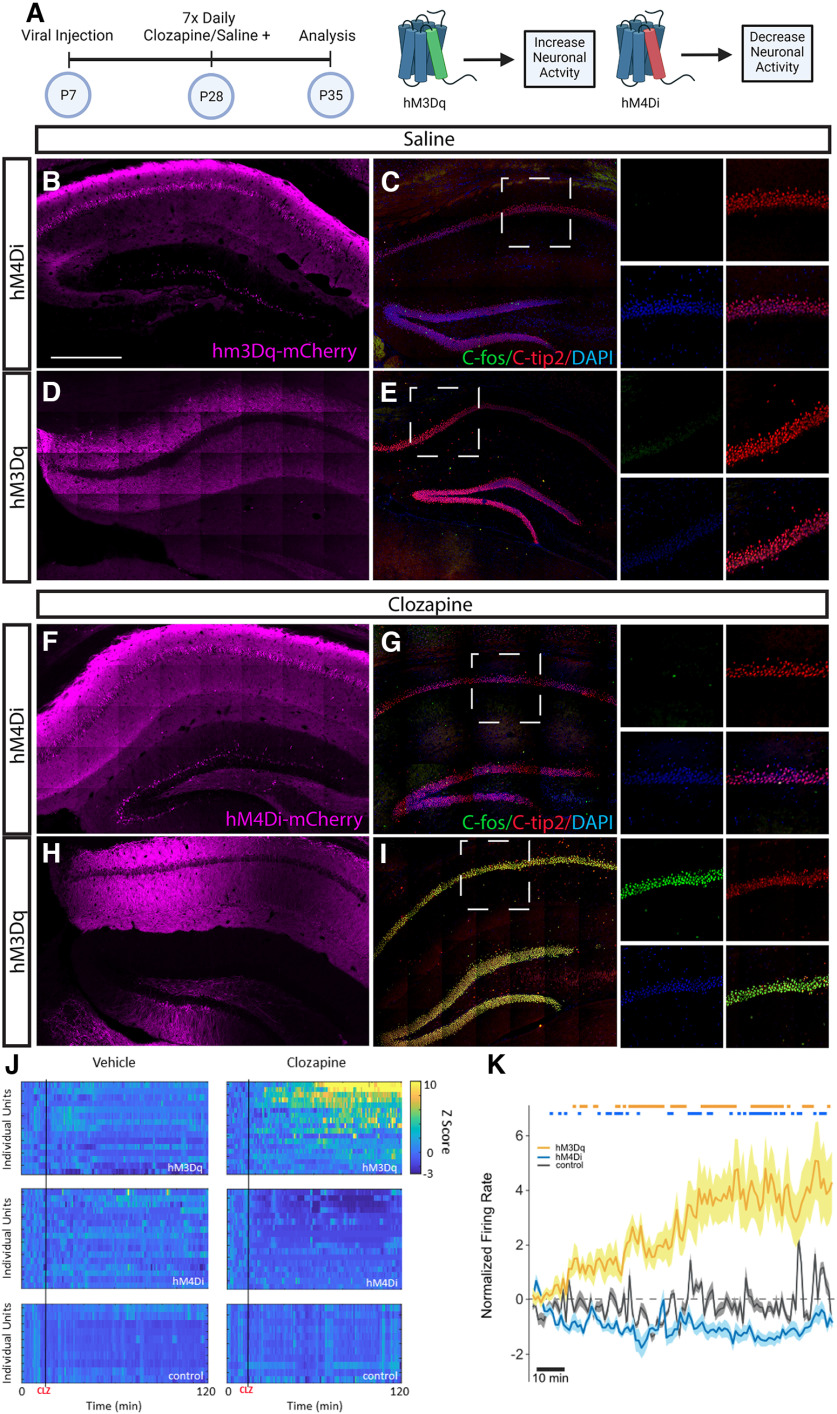
Designer receptors exclusively activated by designer drugs (DREADD) activator Gq results in activation and DREADD activator Gi results in inhibition of the hippocampal neuronal circuit. ***A***, pAAV-hSyn-HA-hM3D(Gq)mCherry or pAAV-hSyn-hM4D(Gi)-mCherry virus were injected into the CA1 of the hippocampus at P7. Three weeks later, animals were treated with seven daily intraperitoneal doses of clozapine or saline. Animals were killed 24 h after the last clozapine dose at P35. ***B***, ***F***, Representative expression of hM4D(Gi)mCherry in the hippocampus. ***D***, ***H***, Representative expression of hM3D(Gq)mCherry in the hippocampus. ***C***, Saline treatment in hM4D(Gi)mCherry-expressing mice or (***E***) saline treatment in hM3D(Gq)mCherry-expressing mice results in no c-fos labeling of Ctip2+ neurons. ***G***, 7-d clozapine treatment in hM4D(Gi)mCherry-expressing mice resulted in low cfos labeling of ctip2+ neurons. ***H***, 7-d clozapine treatment in hM3D(Gq) mCherry-expressing mice resulted in high c-Fos labeling of ctip2+ neurons. ***I***, 7-d clozapine treatment in hM4D(Gi) mCherry-expressing mice resulted in no c-Fos labeling of ctip2+ neurons. ***J***, *In vivo* array recording of cells in CA1 of the hippocampus of mice injected with intraperitoneal clozapine or saline (vehicle). Top histograms, Representative cells recorded from the hippocampus of littermates infected with hM3D(Gq) and injected with saline (left) or clozapine (right). Middle histograms, Representative cells recorded from the hippocampus of littermates infected with hM4D(Gi) and injected with saline (vehicle; left) or clozapine (right). Bottom histograms, Representative cells recorded from the hippocampus of littermates treated with clozapine but no hM4D(Gi) injection (bottom left) or treated with clozapine but no hM3D(Gq) injection (bottom right). ***K***, Average suppression rates from cells in the hippocampus of *N* = 3 mice for each condition on the seventh day of clozapine-treated hM3D(Gq), clozapine-treated hM4D(Gi), or saline-treated control mice. Scale bar: 500 μm. Error bars are standard error of the mean. *Figure Contributions*: Alekhya Parvathaneni and Atehsa Sahagun collected data, Iris Tilton, Alekhya Parvathaneni, and Laura Cocas analyzed data, Laura Cocas and Robert Brock made the figure.

We used chemogenetics to manipulate neuronal activity *in vivo*, targeting CA1 neurons in the hippocampus. We confirmed that injection of pAAV-hSyn-hM3D(Gq)-mCherry or pAAV-hSyn-hM4D(Gi)-mCherry into the CA1 of the hippocampus and subsequent injection of saline did not induce activation of Ctip2+ pyramidal neurons of the hippocampus (Fig. 2*A–E*). Injection of the inhibitory DREADD virus pAAV-hSyn-hM4D(Gi)-mCherry into the CA1 of the hippocampus and subsequent injection of clozapine intraperitoneal for 7 d resulted in no activation of Ctip2+ pyramidal neurons of the hippocampus (Fig. 2*F*,*G*). However, injection of the excitatory DREADD virus pAAV-hSyn-hM3D(Gq)-mCherry into the CA1 of the hippocampus and subsequent injection of clozapine intraperitoneal for 7 d resulted in activation of Ctip2+ pyramidal neurons of the hippocampus, resulting in robust c-Fos expression in these neurons (Fig. 2*H*,*I*). To confirm this effect in real time, and to ensure that the pAAV-hSyn-hM4D(Gi)-mCherry injection with clozapine resulted in neuronal inhibition, as well as to confirm that there were no baseline effects of the DREADDs or clozapine alone, we conducted *in vivo* array recordings to measure the changes in firing rate in individual unit recordings in the CA1 of the hippocampus (Fig. 2*J*). We found that saline in pAAV-hSyn-hM4D(Gi)-mCherry or saline in pAAV-hSyn-hM3D(Gq)-mCherry-injected mice had no effect on neuronal spiking (top left and middle histograms), nor did clozapine treatment alone (bottom histograms), but a 7-d clozapine treatment in pAAV-hSyn-hM4D(Gi)-mCherry mice resulted in inhibition of neuronal activity in the hippocampus, and a 7-d clozapine treatment in pAAV-hSyn-hM3D(Gq)-mCherry-injected mice resulted in robust activation of neuronal activity in the hippocampus (top right and middle histograms and Fig. 2*K*).

**Figure 3.**
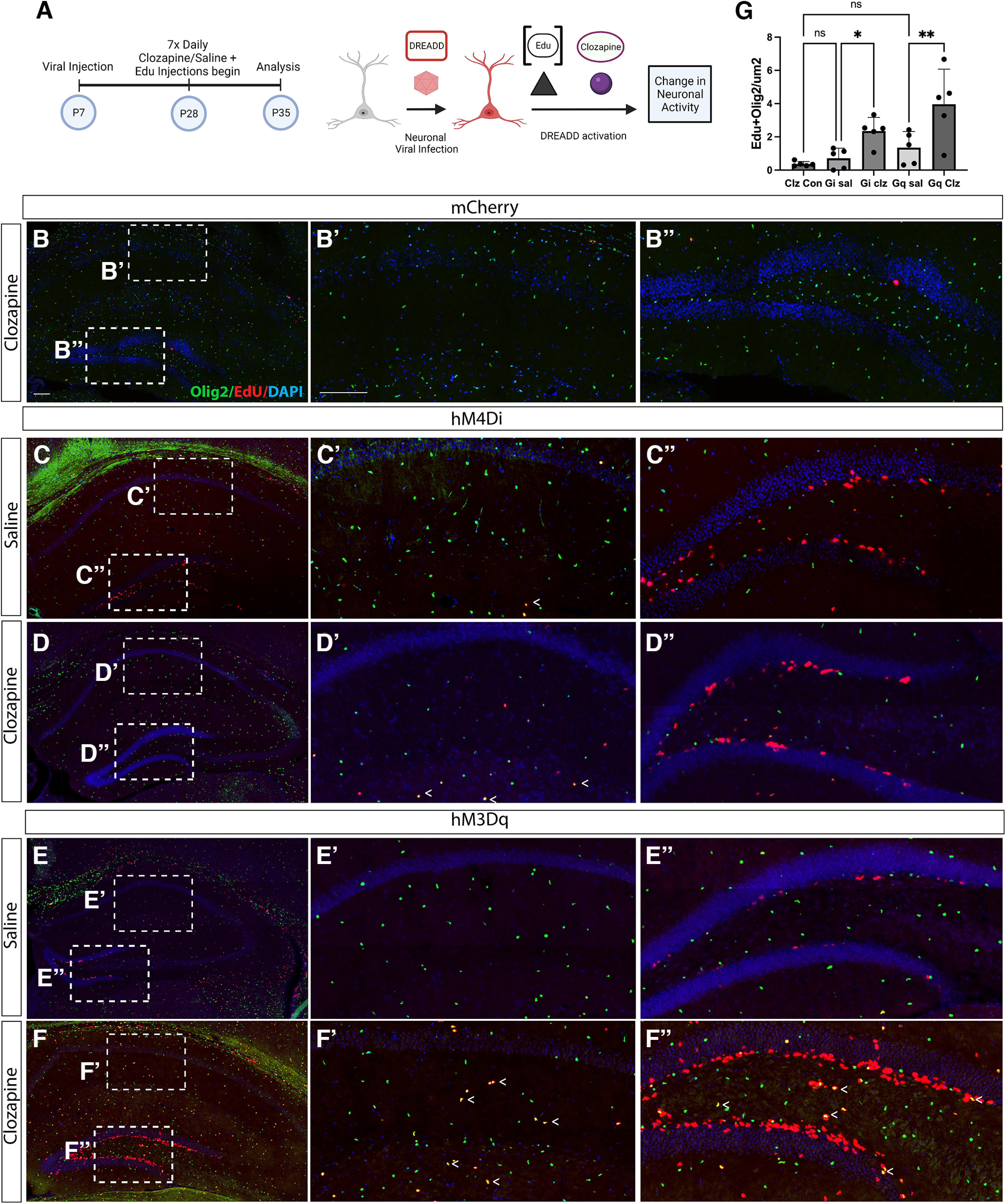
Altering neuronal activity leads to increased cell proliferation in the hippocampus. ***A***, pAAV-hSyn-HA-hM3D(Gq)mCherry or pAAV-hSyn-hM4D(Gi)-mCherry AAV was injected into the CA1 of the hippocampus at P7. Three weeks later, animals were treated with seven daily intraperitoneal doses of Edu plus clozapine or saline. Animals were killed 24 h after the last dose at P35. Confocal images of Edu+ olig2+ OPCs after: (***B***) clozapine treatment in hSyn-mCherry-injected animals, (***C***) saline treatment in hM4Di-injected animals, (***D***) clozapine treatment in hM4Di-injected animals, (***E***) saline treatment in hM3Dq-injected animals, (***F***) clozapine treatment in hM3Dq-injected animals, (***G***) ratio of Edu+/Olig2+ OPCs/100 μm^2^ in clz+ mcherry control, sal+ hM4Di, clz+ hM4Di, sal+ hM3Dq, and clz+ hM3Dq-injected animals (one-way ANOVA compared the effect of neuronal activity on cell proliferation; Fisher’s multiple comparison test was significantly different for Gq sal vs Gq clz, *p* = 0.0016, *t* = 3.65, DF = 20; 95% CI = −4.114 to −1.114 and Gi sal vs Gi clz, *p* = 0.0334, *t* = 2.285, DF = 20; 95% CI = −3.143 to −1.430) NClzCon = 5, mean ClzCon = 0.3661; SEM = 0.0671; NGiSal = 5; mean GiSal = 0.7094; SEM = 0.2728; NGiClz = 5; mean Giclz = 2.352; SEM = 0.3656 NGqSal = 5; mean GqSal = 1.351; SEM = 0.4323; NGqClz = 5; mean Gqclz = 3.966; SEM = 0.9451) Scale bar: 100 μm. Error bars are standard error of the mean. *Figure Contributions*: Atehsa Sahagun, Daniela Moura, Laura Cocas, and Emma Brennan collected data, Daniela Moura and Laura Cocas analyzed data, Daniela Moura, Laura Cocas, and Robert Brock made the figure.

We examined the effect of chronically increased neuronal activity or chronically decreased neuronal activity on OPC development, measuring the number of dividing cells with time-matched Edu and either clozapine or saline treatment (Fig. 3*A*). We hypothesized that decreasing neuronal activity would result in fewer OPCs proliferating, as neuronal activity has been shown to be an important cue for OPC proliferation and differentiation (for review, see Bergles et al., 2010; also, Gallo et al., 1996; Yuan et al., 1998; Gudz et al., 2006). We found this not to be the case: there were more dividing OPCs in the hM4Di(Gi) mice treated with 7 d of clozapine, compared with saline treatment alone (Fig. 3*C*,*D*,*G*). Additionally, we hypothesized that, consistent with previous work (Mitew et al., 2018), increasing neuronal activity would result in increased proliferation of OPCs. We found this to be the case: increasing neuronal activity with 7 d of clozapine treatment in hM3Dq (Gq) mice resulted in an increase in the number of dividing OPCs, compared with saline treatment alone (Fig. 3*E–G*; see Table 1 for additional statistics). This increase in proliferation indicates that there is a larger pool of OPCs available after increasing or decreasing neuronal activity. To confirm that these results were not because of the presence of clozapine, we also injected a pSyn-mcherry virus and treated mice with clozapine. Mice expressing pSyn-mcherry and treated with 7 d of clozapine had no differences in OPC proliferation compared with either hM3D(Gq) mice treated with saline, or hM4D(Gi) mice treated with saline (Fig. 3*B*,*G*).

**Table 1:** Statistical table

Source	Data structure (normality of residuals)	Type of test	Difference between the means	Power (95% CI)
a. Proliferation, Gi Sal vs Gi Clz	Normal distribution	One-way ANOVA	−1.643	−3.143 to −0.1430
b. Proliferation, Gq Sal vs Gq Clz	Normal distribution	One-way ANOVA	−0.9853	−4.114 to −1.114
c. Proliferation, Clz Con vs Gi Sal	Normal distribution	One-way ANOVA	−0.3433	−1.843 to 1.157
d. Proliferation, Clz Con vs Gq Sal	Normal distribution	One-way ANOVA	−2.614	−2.485 to 0.5147
e. Activation, Gi Sal vs Gi Clz	Normal distribution	Unpaired *t* test	0.1226 ± 0.4124	−0.8865 to 1.132
f. Activation, GqSal vs Gq Clz	Normal distribution	Unpaired *t* test	1.979 ± 0.3004	1.244 to 2.714
g. Presynaptic inputs, Gi Sal vs Gi Clz	Normal distribution	Unpaired *t* test	−31.77 ± 36.09	−115.0 to 51.45
h. Presynaptic inputs, Gq Sal vs Gq Clz	Non-normal distribution, *W* = 0.7583	Mann–Whitney	105.4 ± 63.74	−41.58 to 252.4
i. Inhibitory inputs, Gi Sal vs Gi Clz	Non-normal distribution, *W* = 0.7537	Mann–Whitney	0.02900 ± 0.04221	−0.06834 to 0.1263
j. Inhibitory inputs, Gq Sal vs Gq Clz	Non-normal distribution, *W* = 0.7086	Mann–Whitney	−0.02812 ± 0.01858	−0.07097 to 0.01473
k. Excitatory inputs, Gi Sal vs Gi Clz	Normal distribution	Unpaired *t* test	−0.06658 ± 0.06483	−0.2252 to 0.09205
l. Excitatory inputs, Gq Sal vs Gq Clz	Normal distribution	Unpaired *t* test	0.09534 ± 0.04221	−0.007947 to 0.1986

Confidence intervals, distribution, and difference between the means.

**Figure 4.**
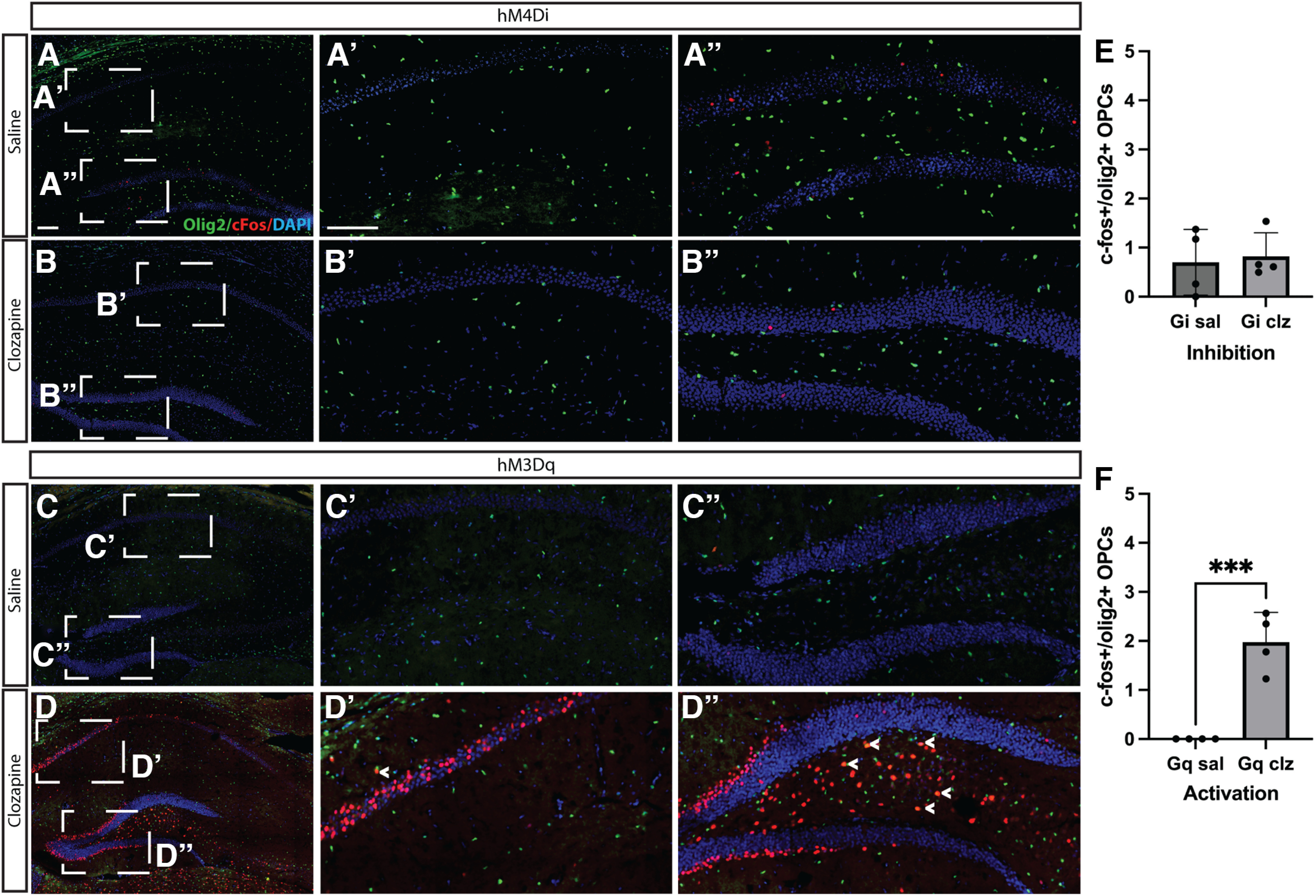
Increasing neuronal activity increases direct activation of OPCs in hippocampus. pAAV-hSyn-HA-hM3D(Gq)mCherry or pAAV-hSyn-hM4D(Gi)-mCherry AAV was injected into the CA1 of the hippocampus at P7. Three weeks later, animals were treated with seven daily intraperitoneal doses of clozapine or saline. Animals were killed 24 h after the last dose at P35. Confocal images of cfos+/olig2+ OPCs after: (***A***) saline treatment in hM4Di-injected animals, (***B***) clozapine treatment in hM4Di-injected animals, (***C***) saline treatment in hM3Dq-injected animals, (***D***) clozapine treatment in hM3Dq-injected animals, (***E***) ratio of activated OPCs/total OPCs in the hippocampus in hM4Di-injected animals [unpaired *t* test (*t* = 0.2973, df = 6) *p* = 0.3811; NGiSal = 4; mean GiSal = 0.0716; SEM = 0.3362; NGiClz = 4; mean Giclz = 0.8242; SEM = 0.2388]. ***F***, Ratio of activated OPCs/total OPCs in hM3Dq-injected animals in the hippocampus [unpaired *t* test (*t* = 2.515, df = 8) *p* = 0.0180; NGqSal = 4; mean GqSal = 0.0002; SEM = 0.0002; NGqClz = 4; mean Gqclz = 1.979; SEM = 0.3004]. Scale bar: 100 μm. Error bars are standard error of the mean. *Figure Contributions*: Atehsa Sahagun and Laura Cocas collected data. Emma Brennan, Daniela Moura, and Laura Cocas analyzed data. Robert Brock, Laura Cocas and Daniela Moura prepared the figure.

We were curious as to whether OPCs themselves were activated by changing neuronal activity, so we stained them with c-Fos, an immediate early gene that is used to measure cell activation in neural cells that is involved in activity-dependent synaptic plasticity (Kovács, 2008). We found that increasing neuronal activity via 7 d of clozapine treatment in hM3D(Gq) led to increased expression of c-Fos in OPCs, consistent with the idea that altering neuronal activity has functional consequences in OPCs (Fig. 4). Decreasing neuronal activity via 7 d of clozapine treatment in the hM4D(Gi) mice did not decrease the number of c-Fos+ OPCs, probably because of the fact that c-Fos expression in OPCs in the saline controls was near baseline, resulting in a floor effect.

### Effect of neuronal activity on neuron to OPC connectivity

**Figure 5.**
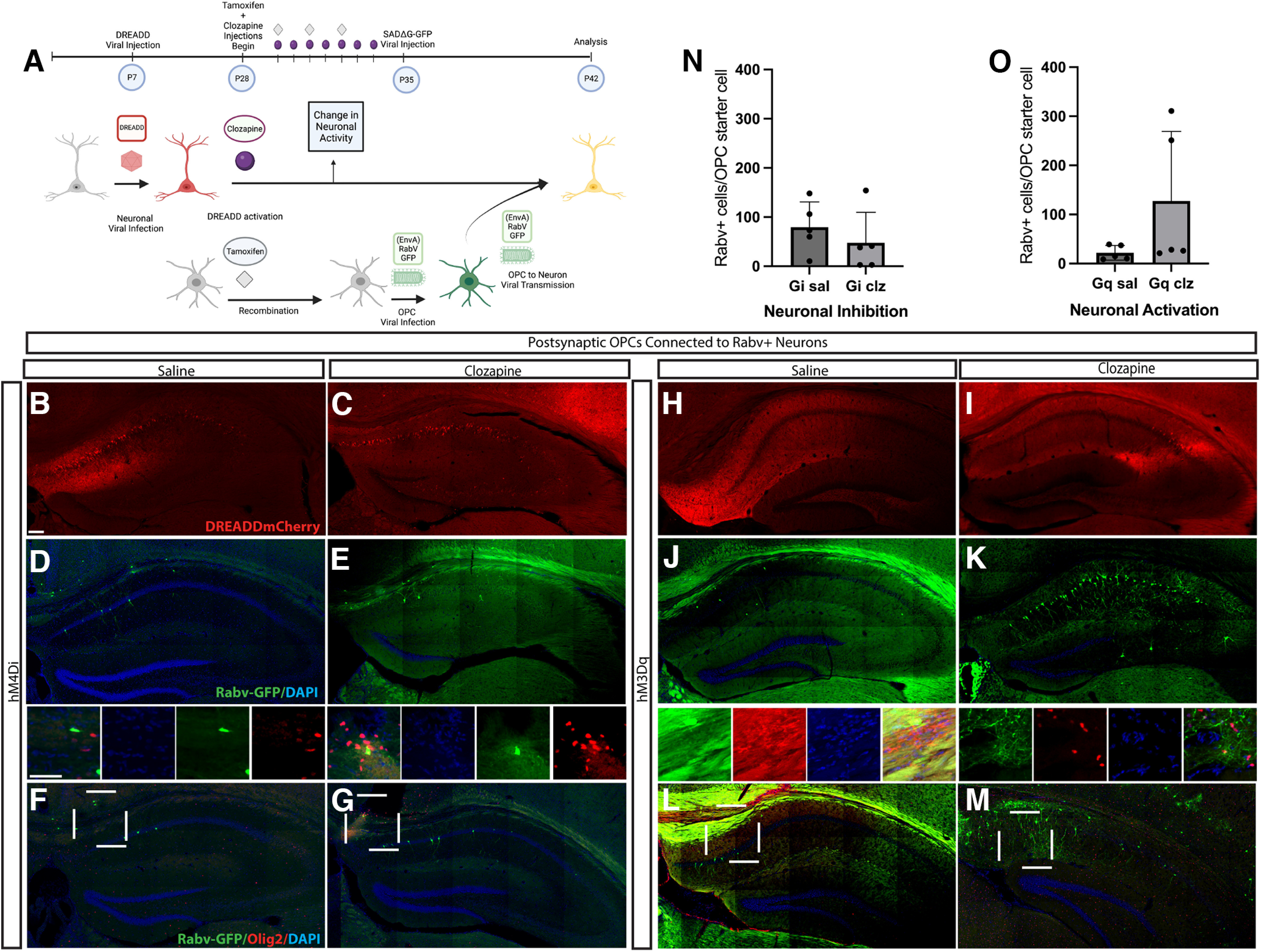
Manipulation of neuronal presynaptic activity onto OPCs does not change total numbers of RabV-labeled neurons connected to OPCs. ***A***, pAAV-hSyn-HA-hM3D-(Gq)mCherry or pAAV-hSyn-hM4D(Gi)-mCherry AAV was injected into the CA1 of the hippocampus at P7. Three weeks later, animals were treated with seven daily intraperitoneal doses of clozapine or saline and four doses of tamoxifen in that 7-d period. Twenty-four hours after the last injection, Pdgfra-^CreERT2^; ROSA^tTA^; pTRE-Bi-G-TVA mice, inducing recombination in OPCs using tamoxifen. We then targeted OPCs with CA1 viral injection of (EnvA)RabV that expresses a green fluorescent reporter (GFP) to measure changes in connections between neurons and OPCs after manipulation of neuronal activity, and stained with Olig2 to label starter cells (GFP+ and Olig2+). In Pdgfra-^CreERT2^; ROSA^tTA^; pTRE-Bi-G-TVA mice, pAAV-hSyn-HA-hM3D-(Gq)mCherry or pAAV-hSyn-hM4D(Gi)-mCherry AAV was injected into the CA1 of the hippocampus at P7. Three weeks later, animals were treated with seven daily intraperitoneal doses of clozapine or saline and four doses of tamoxifen in that 7-d period. Twenty-four hours after the last injection, see then targeted OPCs. ***B***, Red, hM4D(Gi)-mCherry AAV injected in mice treated with saline. ***C***, Red, hM4D(Gi)-mCherry AAV injected in mice treated with clozapine. ***D***, Green/blue, (EnvA)RabV-GFP/DAPI in hM4D(Gi)-injected mice treated with saline. ***E***, Green/blue, (EnvA)RabV-GFP/DAPI in hM4D(Gi)-injected mice treated with clozapine. ***F***, Different section, Green/blue/red, (EnvA)RabV-GFP/DAPI/Olig2 in hM4D(Gi)-injected in mice treated with saline. ***G***, Different section, Green/blue/red, (EnvA)RabV-GFP/DAPI/Olig2 in hM4D(Gi)-injected in mice treated with clozapine. ***H***, Red, hM3D(Gq)-mCherry AAV injected in mice treated with saline. ***I***, Red, hM3D(Gq)-mCherry AAV injected in mice treated with clozapine ***J***, Green/blue, (EnvA)RabV-GFP/DAPI in hM3D(Gq)-injected mice treated with saline. ***K***, Green/blue, (EnvA)RabV-GFP/DAPI in hM3D(Gq)-injected mice treated with clozapine. ***L***, Different section, green/blue/red, (EnvA)RabV-GFP/DAPI/Olig2 in hM3D(Gq)-injected in mice treated with saline. ***M***, Different section, Green/blue/red, (EnvA)RabV-GFP/DAPI/Olig2 in hM3D(Gq)-injected in mice treated with clozapine. ***N***, Number of Rabv+ Cells/Olig2+ OPC Starter Cell hM4D(Gi)-injected mice [unpaired *t* test (*t* = 0.8804, df = 8) *p* = 0.2022; NGiSal = 5; mean GiSal = 79.50; SEM = 23.00; NGiClz = 5; mean GiClz = 47.73; SEM = 27.81]. ***O***, Number of Rabv+ Cells/Olig2+ OPC Starter Cell in hM3D(Gq)-injected mice [Mann–Whitney test (*U* = 6) *p* = 0.1111; NGqSal = 5; mean GqSal = 21.95; SEM = 6.636; NGqClz = 5; mean GqClz = 127.3; SEM = 63.39]. Number of OPCs starter cells RabV+ Olig2+ in: hM4D(Gi)-injected mice: mean GiSal = 46.60; SEM = 42.87; mean GiClz= 11.8; SEM= 8.622; in hM3D(Gq) mice: mean GqSal = 52.20; SEM = 30.72; mean GqClz = 3.2; SEM = 1.114. Scale bar (***B***): 100 um; (***D***) inset above, 50 μm. Error bars are standard error of the mean. *Figure Contributions*: Alekhya Parvathaneni, Atehsa Sahagun, and Laura Cocas collected data. Atehsa Sahagun and Laura Cocas analyzed data. Laura Cocas and Robert Brock prepared the figure.

After finding changes in proliferation and activation of OPCs, we wanted to determine whether there were global changes in connectivity between neurons and OPCs after chronic manipulation of neuronal activity. We combined chemogenetic manipulations of neuronal activity with genetically targeted monosynaptic tracing using deletion mutant rabies virus to determine the changes in presynaptic inputs onto OPCs (Fig. 5*A*). We found that increasing activity via hM3D(Gq) expression and 7 d of clozapine dosing or decreasing neuronal activity via hM4D(Gi) expression and 7 d of clozapine treatment did not change the total number of neuronal inputs onto OPCs (Fig. 5*N–O*). We examined the number of neuronal inputs by region and location within the hippocampus, and by ipsilateral or contralateral inputs, and found no differences (data not shown).

**Figure 6.**
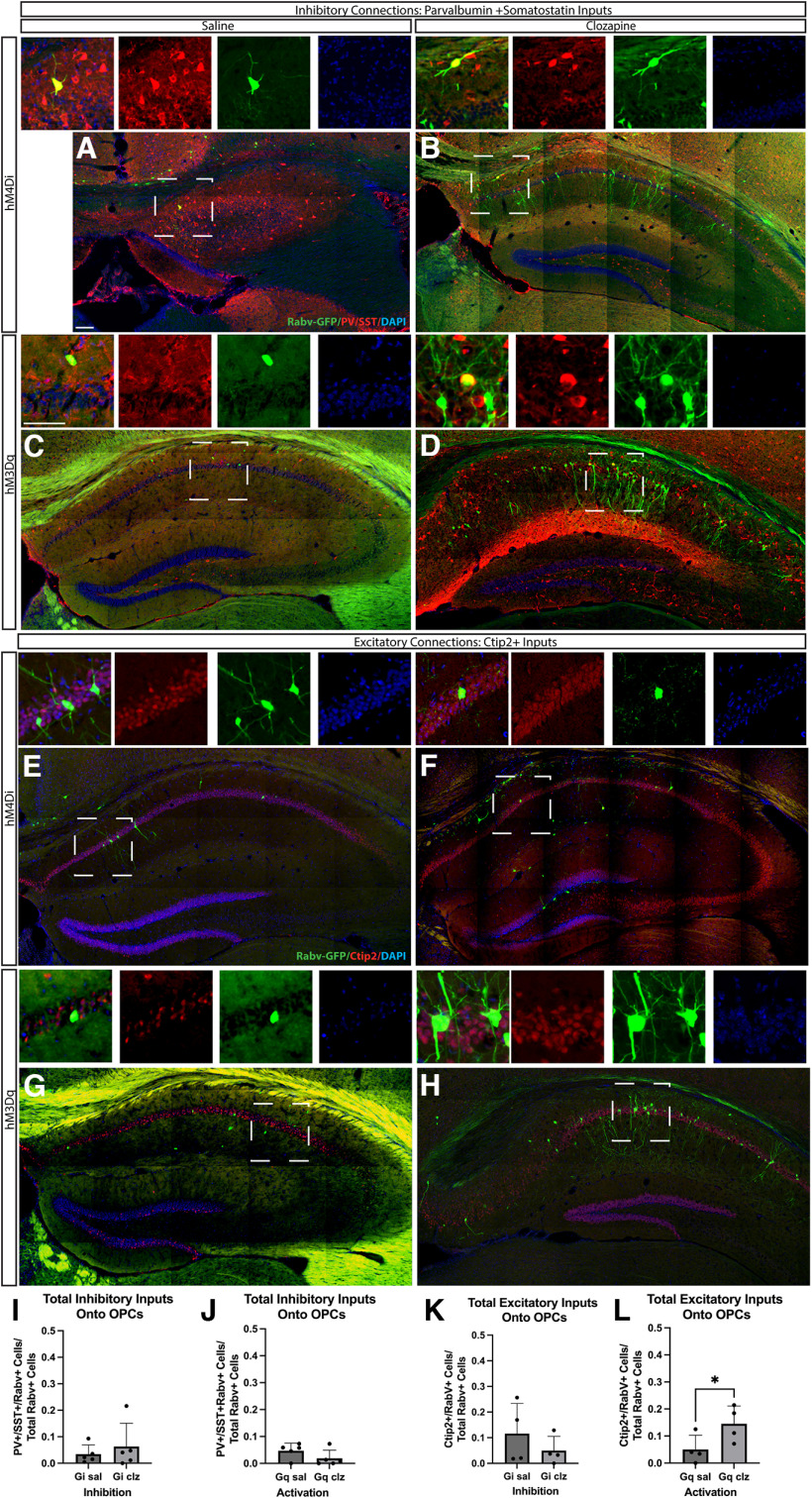
Increasing neuronal activity onto OPCs does not change RabV-labeled inhibitory presynaptic neuronal inputs onto OPCs but increases RabV-labeled excitatory presynaptic inputs onto OPCS. pAAV-hSyn-hM4D(Gi)-mCherry AAV or pAAV-hSyn-HA-hM3D(Gq)mCherry AAV was injected into the CA1 of the hippocampus, treated with seven daily intraperitoneal doses of clozapine or saline, four doses of tamoxifen in Pdgfra-^CreERT2^; ROSA^tTA^; pTRE-Bi-G-TVA mice and then injected with (EnvA)RabV(GFP) to measure changes in connections between neurons and OPCs, then stained with Olig2 and with either PV and SST to measure inhibitory neuron inputs or Ctip2 to measure excitatory neuron inputs. ***A***, Green/blue/red, (EnvA)RabV-GFP/DAPI/PV/SST in hM4D(Gi)-injected in mice treated with saline. ***B***, Green/blue/red, (EnvA)RabV-GFP/DAPI/PV/SST in hM4D(Gi)-injected in mice treated with clozapine. ***C***, Green/blue/red, (EnvA)RabV-GFP/DAPI/PV/SST in hM3D(Gq)-injected in mice treated with saline. ***D***, Green/blue/red, (EnvA)RabV-GFP/DAPI/PV/SST in hM3D(Gq)-injected in mice treated with clozapine. ***E***, Green/blue/red, (EnvA)RabV-GFP/DAPI/Ctip2 in hM4D(Gi)-injected in mice treated with saline. ***F***, Green/blue/red, (EnvA)RabV-GFP/DAPI/Ctip2 in hM4D(Gi)-injected in mice treated with clozapine. ***G***, Green/blue/red, (EnvA)RabV-GFP/DAPI/Ctip2 in hM3D(Gq)-injected in mice treated with saline. ***H***, Green/blue/red, (EnvA)RabV-GFP/DAPI/Ctip2 in hM3D(Gq)-injected in mice treated with clozapine. ***K***, % of Ctip2+ neurons connected to OPCs after neuronal inhibition [unpaired *t* test (*t* = 1.027, df = 6) *p* = 0.1720; NGiSal = 4; mean GiSal = 11.162; SEM = 5.861; NGiClz = 4; mean GiClz = 4.965; SEM = 2.711]. ***L***, % of Ctip2+ neurons connected to OPCs after neuronal excitation [unpaired *t* test (*t* = 2.259, df = 6) *p* = 0.0323; NGqSal = 4; mean GqSal = 5.001; SEM = 2.654; NGqClz = 4; mean GqClz = 14.53; SEM = 3.282]. ***I***, % of PV/SST neurons connected to OPCs after neuronal inhibition [Mann–Whitney test (*U* = 11) *p* = 0.8413; NGiSal = 5; mean GiSal = 3.424; SEM = 1.552; NGiClz = 5; mean GiClz = 6.324; SEM = 3.926]. ***J***, % of PV/SST neurons connected to OPCs after neuronal excitation [Mann–Whitney test (*U* = 7) *p* = 0.3016; NGqSal = 5; mean GqSal = 4.701; SEM = 1.255; NGqClz = 5; mean GqClz = 1.889; SEM = 1.370]. Scale bar (***A***) 100 μm, (***C***, inset) 50 μm. Error bars are standard error of the mean. *Figure Contributions*: Alekhya Parvathaneni, Atehsa Sahagun, and Laura Cocas collected data. Alekhya Parvathaneni and Laura Cocas analyzed the data. Laura Cocas made the figure.

We hypothesized that the total number of neuronal inputs might be unaffected, but the specific types of neurons that were connected to OPCs would be altered by changes in neuronal activity. However, decreasing neuronal activity did not lead to changes in RabV-labeled inhibitory inputs: there was no difference in the number of SST+/PV+ inhibitory neurons connected to OPCs in Hm4Di-expressing mice after 7 d of clozapine treatment compared with saline controls (Fig. 6*A*,*B*,*I*). The number of RabV-labeled excitatory inputs onto OPCs also did not change with a decrease in neuronal activity: we saw no difference in the number of Ctip2+ excitatory connections onto OPCS after 7 d of clozapine treatment in Hm4D(Gi)-expressing mice compared with saline controls (Fig. 6*E*,*F*,*K*). Interestingly, increasing neuronal activity mice led to an increase in the number of RabV-labeled excitatory inputs connected to OPCs: we found that the number of Ctip2+ excitatory neurons connected to OPCs increased in Hm3Dq mice treated with 7 d of clozapine compared with saline controls (Fig. 6*G*,*H*,*L*). We found that after increasing neuronal activity the number of RabV-labeled inhibitory inputs onto OPCs was unaffected: we saw no difference in the number of PV+/SST+ connections onto OPCS after 7 d of clozapine treatment in Hm4D(Gi)-expressing mice compared with saline controls (Fig. 6*C*,*D*,*J*). These results indicate that modulating neuronal activity can impact neuronal glial connectivity.

## Discussion

Our understanding of how neuronal activity regulates synaptic inputs from neurons onto OPCs, and whether this is instructive for OPC target selection of neuronal axons and subsequent myelination patterns, is still very incomplete. In order to elucidate this, we targeted OPCs in different forebrain regions to understand the extent that neurons contact OPCs in regionally specific ways. We also manipulated neuronal activity to investigate the effect of neuronal activity on OPC responses, specifically examining OPC proliferation and OPC activation. Finally, we examined whether neuronal connections onto OPCs are directly altered by changing the tuning of neuronal excitability. We are following up on OPC target selection and subsequent myelination patterns to determine whether the neuronal activity is instructive for these processes in an additional manuscript in preparation (unpublished data, Daniela Moura and Laura Cocas).

Circuits in the cerebral cortex, hippocampus, and striatum are formed by different populations of neurons that integrate input from both local and distant input regions. It is expected that the pattern of myelination will follow a diverse distribution, and is correlated with the activity of each region. Moreover, neuronal subtypes in the cortex exhibit diverse myelination profiles (Tomassy et al., 2014; Call and Bergles, 2021). We found that the patterns of these connections were consistent with the expected local circuitry and there were differences in the number of inputs and proportion of inhibitory and excitatory connections. Similar data has been described before by Mount et al. (2019) in different brain regions such as corpus callosum, premotor cortex, and primary somatosensory cortex. We expanded that analysis to include the dorsal striatum and hippocampus, and it was important for us to investigate which of the systems we would focus our analysis on synaptic contact. To explore this in greater detail, we analyzed whether there were differences in inputs from different neuronal subtypes and brain regions. We examined the distribution of excitatory neuronal inputs with NeuN, which preferentially marks excitatory synapses, and the distribution of inhibitory inputs with PV and SST, the two major subclasses of inhibitory interneurons in the forebrain. In this work we focused on anatomic analyses of cellular subtypes and viral circuit tracing to describe the number and types of connections between OPC and neurons; future work manipulating neuronal activity combined with paired electrophysiological recordings are important to examine functional changes in OPCs as a result of changes in neuronal activity. It is intriguing that for all brain regions, neuronal inputs to OPCs followed the patterns of local cytoarchitecture, suggesting that neuron to OPC connectivity follows programs to build connected networks of cells similar to neuronal networks. The number of neuron to OPC inputs was a magnitude of order smaller: thousands of granule neurons were connected to each Purkinje cell in the cerebellum, and ∼600 long distance and 20 local neurons were connected to each PV+ IN in the cortex (Wall et al., 2010). This difference is likely because of a real difference in the number of OPC connections, but also to differences in viral targeting. In contrast to previous studies, we addressed this issue by not using helper viruses to deliver the TVA or G, nor did we use the ROSA-TVA/G mouse. Instead, we used a two-step tTA-dependent activation of a bicistronic Tet dependent construct that allowed production of TVA and G solely in OPCs. This method was selected to reduce the possibility for recombination in cells other than OPCs, as any neuronal recombination would profoundly impact our results.

We expected that the proliferation rates would be increased in the excitatory stimulation group as described in the literature (Bergles et al., 2010), but we observed a significant difference in both groups. We are confident that our chemogenetic approach specifically targeted Ctip2+ neurons in the hippocampus (Fig. 2), so it is unlikely that this is because of an effect of GABAergic-to-OPC synapses. We also analyzed the effect of clozapine alone, in case our manipulations were leading to generalized OPC proliferation, and found no difference between clozapine alone and our other controls (Fig. 3). However, there is conflicting data regarding the role of excitation on OPC proliferation: some groups have found that increasing AMPA receptors (AMPARs) has been shown not to induce an increase in proliferation (Chen et al., 2018; Stedehouder et al., 2018); others have found that AMPAR gain of function experiments can increase proliferation (Gudz et al., 2006). The mechanism by which neuronal inhibition leads inducing an increase in OPC proliferation is not known. The existing literature suggests that inhibition can, but does not always, lead to increased proliferation: GABA either had no influence on OPC proliferation or led to an increase in proliferation (Balia et al., 2017 vs Hamilton et al., 2017). Other work also found an increase in oligodendrocyte proliferation after loss of input (Mangin et al., 2012; Sahel et al., 2015). Further, the mechanism of lesions and demyelination may seem very different from chemogenetic silencing, but Maldonado and Angulo (2015) suggest a participation of calcium signaling independent from synaptic inputs that may play an important role in modulating OPC proliferation. Examining downstream calcium signaling may therefore help to resolve these conflicting results regarding the role of neuronal activity on OPC proliferation.

It is unknown why some neurons, but not others, exhibit activity-regulated myelination: there may be more than one neuronal population that we are capturing with our chemogenetic approach (neurons that undergo activity dependent myelination and those that do not). Almeida et al. (2021) imaged synaptic vesicle fusion in individual neurons in living zebrafish and observed a feedforward model, where they show that axonal vesicular fusion was enriched in hotspots and promoted and consolidated nascent sheath growth. Therefore, the onset of myelination promoted localized axonal vesicular fusion that in turn promoted myelin growth, as well as a higher likelihood for the OPC to respond to specific neuronal synapses. While we did not test the latter in these experiments, our data are consistent with these findings.

The timing of our experiments is likely relevant to the observed effects, as our activity-dependent manipulations were conducted between P28 and P35, in juvenile animals where synapses, circuits, and myelination are still undergoing substantial modification, but after the initial peak in developmental synapse formation, OPC proliferation, and myelination. The plasticity mechanisms may look very different at P14, during this initial phase of synapse formation and myelination, or at P7, when OPC proliferation is at its peak. Examining these earlier stages in future studies will be valuable in exploring the role of developmental plasticity in neuron to OPC synapse formation and circuit formation.

We expected that neuronal activity would play a role in the modification of neuron to OPC connections labeled by viral tracing. Specifically, our hypothesis was that there would be an increase in connections between neurons and OPCs when neuronal activity was increased and a decrease in connections when neuronal activity was inhibited. Contrary to our expectations, we did not observe any significant differences in the total number of neuron to OPC synapses when neuronal activity was increased or decreased. It is possible that it is not the number of neurons connected to OPCs, but the strength of those connections, that is altered by neuronal activity: future electrophysiological studies will be necessary to determine this. We did find a change in the subtypes of neuronal inputs that were connected to OPCs. Moreover, there was no significant change in the number of INs connected to OPCs, regardless of whether neuronal activity was increased or decreased. This may have been because of the smaller number of INs connected to OPCs to begin with in the hippocampus; perhaps if we had examined IN inputs to OPCs in the cortex, where there is a larger percentage of IN to OPC connections, we would have found a more robust effect. Importantly, we did find a difference in the number of Ctip2+ excitatory neuron inputs onto OPCs when neuronal activity was increased. This was not true when neuronal activity was decreased, which suggests that neuronal activity is permissive for neuronal connectivity onto OPCs but is not necessary: connections still form even when neurons are silenced. However, It is also possible that it is harder to detect changes in inhibitory inputs onto OPCs, since the total number of inhibitory inputs are fewer onto OPCs relative to the number of excitatory inputs. The change in the balance of excitatory input is consistent with the idea that regulating the number of connections onto OPCs is a mechanism for plasticity and has functional consequences in OPCs. Indeed, we find that increasing neuronal activity results in more activated OPCs, measured by c-Fos labeling. These data suggest that like neurons, OPCs may undergo synaptic plasticity as a result of changes in neuronal excitability; additional functional experiments are required to prove this. We are following up on the role of plasticity in myelination and in myelin node length in a separate study.

It was surprising to find that increasing neuronal activity led to an increase in the number of excitatory inputs onto OPCs, but no significant difference in the total number of neurons; more neurons would be expected if the total number of inputs were altered. However, Ctip2, our excitatory neuronal marker, primarily marks excitatory neurons in the pyramidal layers of the hippocampus, and PV and SST, the inhibitory neuron markers we used, mark two of the major subclasses of inhibitory interneurons but not all inhibitory neurons. It is possible that there is a change in the number of CB+ neurons in the dentate gyrus, for example, or VIP+ interneurons in CA1, that are undescribed here. Further characterization of all of the subtypes of neurons that connect to OPCs would help elucidate the composition of RabV+ neurons that were not labeled by Ctip2, PV, or SST. It is also important to note the caveats of viral circuit tracing, which is not a functional approach. Previous work has shown that viral circuit tracing captures synaptic connections, via complementing tracing with electrophysiological recordings (Wall et al., 2010). However, we do not know anything about the strength of these connections, nor whether any of these connections are silent synapses. Electrophysiological experiments will be necessary to address these important functional questions in the future.

Finally, we found that in two of our four RabV experimental groups, there was at least one animal in which the total number of neuron to OPC synapses were an order of magnitude higher. It is unclear why this occurred since all animals were treated equally, dosed with the same tamoxifen, were age matched, received the same dose of DREADD virus and Rabies virus, counterbalanced for sex, and killed the same number of days after the Rabies virus injection. It is possible that there is a small subpopulation of OPCs that are more highly connected to neurons, and we may occasionally target one of these OPCs when sampling from this heterogeneous population of OPCs. As this event is rare, it is difficult to postulate what the function of these “super connected” OPCs are. However, even with these superconnectors removed from the sample, we found no differences in the total number of neuronal inputs to OPCs when neuronal activity was increased or decreased.

Synaptic communication between neurons and OPCs shares common features found in neuron to neuron synapses, such as inhibitory and excitatory postsynaptic receptors, scaffolding proteins, and ion channels expressed in OPCs. In addition to measuring synaptic inputs onto OPCs, future experiments measuring postsynaptic scaffolding proteins, and GABA, AMPA receptors and NMDA receptors would provide valuable insight into postsynaptic changes in OPCs after changing neuronal activity (Arellano et al., 2016; Chen et al., 2018). The question is whether this response is consistent with the sign of neuronal activation: does increased neuronal activation lead to increased excitatory synapses onto OPCs? Or does increased activation result in a compensatory increase in inhibitory synapses? The composition of excitatory and inhibitory synapses onto an individual OPC is not well understood, nor is it understood how neuronal activity regulates receptor expression or turnover directly. It is clear that plasticity in OPCs is a Ca2+ dependent process (Cheli et al., 2016) and that ion channels and neurotransmitters are highly dynamic in these cells (Spitzer et al., 2019). Future work examining the specific receptor turnover that occurs in response to neuronal activity will be important to expand our understanding of plasticity in OPCs.

In summary, our findings contribute to a better understanding of the mechanisms of OPC development and circuit formation during juvenile myelination in response to neuronal activation. This study has the potential to direct research toward therapeutic approaches to treat demyelinating diseases, as adaptive myelination and remyelination likely follow similar mechanisms. For example, manipulating neuronal activity may have the potential to alter myelination in specific regions and different systems, via increasing OPC cell proliferation. But there is still much to explore: the downstream signaling pathways that are activated in OPCs after neuronal activity modulation, the changes in myelination, and the role of synaptic activity in regulating myelination programs. Future work will shed light on these mechanisms.

## Synthesis

Reviewing Editor: Nathalie Ginovart, Universite de Geneve Decisions are customarily a result of the Reviewing Editor and the peer reviewers coming together and discussing their recommendations until a consensus is reached. When revisions are invited, a fact-based synthesis statement explaining their decision and outlining what is needed to prepare a revision will be listed below. The following reviewer(s) agreed to reveal their identity: Matthew Swire, Wenjing Sun.

The authors addressed the main concerns raised through the first review process with a number of experiments that have strengthened the manuscript. However, both reviewers agree that the conclusions drawn from the data collected require some modifications before publication.

Please find the specific comments and queries raised by the reviewers below:

Reviewer 2:

This manuscript utilizes state-of-the-art retrograde monosynaptic labeling techniques with rabies virus tracing to investigate the neuron populations that make synaptic contacts with oligodendrocyte precursor cells (OPCs) during early postnatal development from multiple brain regions. Moreover, the authors used chemogenetic tools to manipulate neuronal activities in the mouse hippocampus and reported corresponding changes in OPC proliferation and the number of neurons that make synapses with OPCs.

This revised manuscript added several sets of important control experiments to address first-round review criticisms and to rule out the possibility of non-specific clozapine or tamoxifen effects. The authors also added more information regarding the statistical analysis and viral tracing technical details. These revisions definitely strengthened the manuscript. The finding that enhanced neuronal activity led to an increase in the number of excitatory neurons making synapses with OPCs is very intriguing and will provide further insights into our understanding of activity-dependent myelination. In my opinion, the work in the current format can stand on its own without requiring additional experiments.

However, I do have some comments/reservations regarding some wording in the manuscript, particularly the conclusions that the authors drew from their experiments:

1. I think the authors would agree that this manuscript is a purely histological characterization of rabies virus-labeled pre-synaptic neurons. While I am very enthusiastic about the results that the number of excitatory pyramidal neurons, not PV+ or SST+ interneurons, making synapses with OPCs, increased after activating hippocampal neuronal activity with Gq DREADD, I think it is a far stretch and overselling to claim that “neuronal activity altered the number of neuronal-glial synaptic connections” or “neuronal activity alters excitatory neuron to OPC synaptic inputs”. The results only showed the changes in the number of pyramidal neurons that are synaptically connected with OPCs, but we do not know how many synapses each connecting neuron makes with OPCs. We also don’t know whether these synaptic contacts are functioning or silent, or how neuronal activity alters the synaptic strength of these synapses. Answering these questions will require thorough physiological characterization and will be beyond the scope of the current study. Therefore, I would suggest the authors carefully revise the conclusions throughout different parts of the manuscript, to better reflect the actual results and significance of the study. I would also suggest authors include additional discussion on the limitation of their histological approach in investigating the alteration in neuron-OPC synaptic connections.

2. Similarly, it is very interesting to see that increasing neuronal activity led to increased c-Fos+ OPCs. However, I disagree with authors’ claim that c-Fos is used to measure synaptic plasticity, or is considered a synaptic plasticity marker. c-Fos is indeed a marker for activated cells. More activated OPCs could also be explained by the more frequent release of synaptic vesicles due to increased pre-synaptic neuronal firing. Instead, synaptic plasticity strictly refers to the changes in the strength of synaptic transmission. This study in the current format does not provide any evidence about the alteration in either the total number of synapses or the synaptic strength.

3. The authors reported that not only did increasing neuronal activities increase the proliferation of OPCs, but decreasing neuronal activity with inhibitory Gi also led to more dividing OPCs. This unexpected result somehow contradicts their hypothesis. The authors should add some discussion about this result and provide their interpretation and perspective on that.

Some minor errors:

a) Repeated sentences between line 369 - 381

b) Line 322, the correct corresponding figure panel should be Figure 1N, not Figure 1O

c) Figure 1N, the annotation for boxes with different patterns are missing in both figure panel and legends. I assume they refer to excitatory, PV+ and SST+ neurons?

d) Figure for c-Fos staining, in hM3Dq panel, it looks like the dashed boxes referring to C’ and C’ and D’ and D’are missing.

## Author Response

Reviewer comments and responses:

1. I think the authors would agree that this manuscript is a purely histological characterization of rabies virus-labeled pre-synaptic neurons. While I am very enthusiastic about the results that the number of excitatory pyramidal neurons, not PV+ or SST+ interneurons, making synapses with OPCs, increased after activating hippocampal neuronal activity with Gq DREADD, I think it is a far stretch and overselling to claim that “neuronal activity altered the number of neuronal-glial synaptic connections” or “neuronal activity alters excitatory neuron to OPC synaptic inputs”. The results only showed the changes in the number of pyramidal neurons that are synaptically connected with OPCs, but we do not know how many synapses each connecting neuron makes with OPCs. We also don’t know whether these synaptic contacts are functioning or silent, or how neuronal activity alters the synaptic strength of these synapses. Answering these questions will require thorough physiological characterization and will be beyond the scope of the current study. Therefore, I would suggest the authors carefully revise the conclusions throughout different parts of the manuscript, to better reflect the actual results and significance of the study. I would also suggest authors include additional discussion on the limitation of their histological approach in investigating the alteration in neuron-OPC synaptic connections.

R- We’ve addressed the reviewer’s concerns by rephrasing our claims throughout the manuscript.

The title is now:

Neuronal Activity Changes the Number of Neurons that are Synaptically Connected to OPCs

Abstract (Page 1):

Modulation of neuronal activity also altered the organization of neuronal-glial connections: while it did not impact the total number of RabV-labeled neuronal inputs, or the number of RabV-labeled inhibitory neuronal inputs, it did alter the number of RabV-labeled excitatory neuron to OPC connections.

Last paragraph in the results section (page 10):

However, decreasing neuronal activity did not lead to changes in RabV-labeled inhibitory inputs: there was no difference in the number of SST+/PV+ inhibitory neurons connected to OPCs in Hm4Di-expressing mice after 7 days of clozapine treatment compared to saline controls (Figure 6A-B and I). The number of RabV-labeled excitatory inputs onto OPCs also did not change with a decrease in neuronal activity: we saw no difference in the number of Ctip2+ excitatory connections onto OPCS after 7 days of clozapine treatment in Hm4D(Gi) expressing mice compared to saline controls (Figure 6E-F and K). Interestingly, increasing neuronal activity mice led to an increase in the number of RabV-labeled excitatory inputs connected to OPCs: we found that the number of Ctip2+ excitatory neurons connected to OPCs increased in Hm3Dq mice treated with 7 days of clozapine compared to saline controls (Figure 6G-H and L). We found that after increasing neuronal activity the number of RabV-labeled inhibitory inputs onto OPCs was unaffected: we saw no difference in the number of PV+/SST+ connections onto OPCS after 7 days of clozapine treatment in Hm4D(Gi) expressing mice compared to saline controls (Figure 6 C-D and J). These results indicate that modulating neuronal activity can impact neuronal glial connectivity.

The second paragraph of the discussion section (page 11):

In this work we focused on anatomical analyses of cellular subtypes and viral circuit tracing to describe the number and types of connections between OPC and neurons; future work using paired electrophysiological recordings are important to examine functional changes in OPCs.

The sixth paragraph of the discussion section (page 12):

We expected that neuronal activity would play a role in the modification of neuron to OPC connections labeled by viral tracing. Specifically, our hypothesis was that there would be an increase in connections between neurons and OPCs when neuronal activity was increased and a decrease in connections when neuronal activity was inhibited. Contrary to our expectations, we did not observe any significant differences in the total number of neuron to OPC synapses when neuronal activity was increased or decreased. It is possible that it is not the number of neurons connected to OPCs, but the strength of those connections, that is altered by neuronal activity: future electrophysiological studies will be necessary to determine this.

The seventh paragraph of the discussion section (page 13)

It is also important to note the caveats of viral circuit tracing, which is not a functional approach. Previous work has shown that viral circuit tracing captures synaptic connections, via complementing tracing with electrophysiological recordings (Wall, et al., 2010). However, we do not know anything about the strength of these connections, nor whether any of these connections are silent synapses. Electrophysiological experiments will be necessary to address these important functional questions in the future.

In the conclusion section (page 13):

In summary, our findings contribute to a better understanding of the mechanisms of OPC development and circuit formation during juvenile myelination in response to neuronal activation

2. Similarly, it is very interesting to see that increasing neuronal activity led to increased c-Fos+ OPCs. However, I disagree with authors’ claim that c-Fos is used to measure synaptic plasticity, or is considered a synaptic plasticity marker. c-Fos is indeed a marker for activated cells. More activated OPCs could also be explained by the more frequent release of synaptic vesicles due to increased pre-synaptic neuronal firing. Instead, synaptic plasticity strictly refers to the changes in the strength of synaptic transmission. This study in the current format does not provide any evidence about the alteration in either the total number of synapses or the synaptic strength.

Response - We’ve changed the c-Fos results explanation.

In the last paragraph of the section “Manipulation of Neuronal Activity to Test Changes in OPC Function” (page 9):

We were curious as to whether OPCs themselves were activated by changing neuronal activity, so we stained them with c-Fos, an immediate early gene that is used to measure cell activation in neural cells that is involved in activity-dependent synaptic plasticity (Kovacs, et al., 2008).

3. The authors reported that not only did increasing neuronal activities increase the proliferation of OPCs, but decreasing neuronal activity with inhibitory Gi also led to more dividing OPCs. This unexpected result somehow contradicts their hypothesis. The authors should add some discussion about this result and provide their interpretation and perspective on that. -

R- We’ve expanded our explanation of this topic.

In the third paragraph in the discussion section (page 11):

However, there is conflicting data regarding the role of excitation on OPC proliferation: some groups have found that increasing AMPARs has been shown not to induce an increase in proliferation (Chen et al 2018; Stedehouder et al., 2018); others have found that AMPAR gain of function experiments can increase proliferation (Gudz, et al., 2006). The mechanism by which neuronal inhibition leads to an increase in OPC proliferation is not known. The existing literature suggests that inhibition can, but does not always, lead to increased proliferation: GABA either had no influence on OPC proliferation or led to an increase in proliferation (Balia et al., 2017 vs. Hamilton, et al., 2017). Other work also found an increase in oligodendrocyte proliferation after loss of input (Mangin et al., 2012; Sahel et al., 2015). Further, the mechanism of lesions and demyelination may seem very different from chemogenetic silencing, but Maldonado and Maria (2014) suggest a participation of calcium signaling independent from synaptic inputs that may play an important role in modulating OPC proliferation. Examining downstream calcium signaling may therefore help to resolve these conflicting results regarding the role of neuronal activity on OPC proliferation.

And also in the fifth paragraph of the discussion section (page 12):

This was not true when neuronal activity was decreased, which suggests that neuronal activity is permissive for neuronal connectivity onto OPCs but is not necessary: connections still form even when neurons are silenced. However, It is also possible that it is harder to detect changes in inhibitory inputs onto OPCs, since the total number of inhibitory inputs are fewer onto OPCs relative to the number of excitatory inputs. The change in the balance of excitatory input is consistent with the idea that regulating the number of connections onto OPCs is a mechanism for plasticity and has functional consequences in OPCs. Indeed, we find that increasing neuronal activity results in more activated OPCs, measured by c-fos labeling. These data suggest that like neurons, OPCs may undergo synaptic plasticity as a result of changes in neuronal excitability; additional functional experiments are required to prove this.

Some minor errors:

a) Repeated sentences between line 369 - 381

R - We’ve removed the repeated sentence.

b) Line 322, the correct corresponding figure panel should be Figure 1N, not Figure 1O

R - We’ve changed the wrong legend letter.

c) Figure 1N, the annotation for boxes with different patterns are missing in both figure panel and legends. I assume they refer to excitatory, PV+ and SST+ neurons?

R-We have added the legend which was accidentally hidden by the other panel. The annotations are also present in the legend.

d) Figure for c-Fos staining, in hM3Dq panel, it looks like the dashed boxes referring to C’ and C’ and D’ and D’are missing.

R-We have added the missing dashed boxes.

We also corrected this sentence on the second to last paragraph on the results section topic “Manipulation of Neuronal Activity to Test Changes in OPC Function” (page 9)

Mice expressing pSyn-mcherry and treated with 7 days of clozapine had no differences in OPC proliferation compared to either hM3D(Gq) mice treated with saline, or hM4D(Gi) mice treated with saline (Figure 3B,G).
